# Order Matters: The Benefits of Ordinal Fragility Curves for Damage and Loss Estimation

**DOI:** 10.1111/risa.13815

**Published:** 2021-08-23

**Authors:** Michele Nguyen, David Lallemant

**Affiliations:** ^1^ Asian School of the Environment Nanyang Technological University Singapore; ^2^ Earth Observatory of Singapore Nanyang Technological University Singapore

**Keywords:** Building damage, fragility function, generalized linear model, ordinal regression, seismic vulnerability

## Abstract

Probabilistic loss assessments from natural hazards require the quantification of structural vulnerability. Building damage data can be used to estimate fragility curves to obtain realistic descriptions of the relationship between a hazard intensity measure and the probability of exceeding certain damage grades. Fragility curves based on the lognormal cumulative distribution function are popular because of their empirical performance as well as theoretical properties. When we are interested in estimating exceedance probabilities for multiple damage grades, these are usually derived per damage grade via separate probit regressions. However, they can also be obtained simultaneously through an ordinal model which treats the damage grades as ordered and related instead of nominal and distinct. When we use nominal models, a collapse fragility curve is constructed by treating data of “near‐collapse” and “no damage” the same: as data of noncollapse. This leads to a loss of information. Using synthetic data as well as real‐life data from the 2015 Nepal earthquake, we provide one of the first formal demonstrations of multiple advantages of the ordinal model over the nominal approach. We show that modeling the ordering of damage grades explicitly through an ordinal model leads to higher sensitivity to the data, parsimony and a lower risk of overfitting, noncrossing fragility curves, and lower associated uncertainty.

## 1 INTRODUCTION

Loss assessment from natural hazards requires the quantification of vulnerability of exposed infrastructure. Fragility curves address this by relating a hazard intensity measure (IM) to the probability that a building or structure exceeds a certain damage grade. These relationships can be defined via expert judgment (Applied Technology Council, 1985; Jaiswal, Aspinall, & Perkins, 2012), structural analysis (Federal Emergency Management Agency, 1999; Ibarra & Krawinkler, 2005; Rossetto & Elnashai, 2005; Singhal & Kiremidjian, 1996), or derived using empirical damage data (Braga, Dolce, & Liberatore, 1982; Colombi et al., 2008; Lantada et al., 2009; Sabetta, Goretti, & Lucantoni, 1998). While the first approach relies heavily on the expertise of the selected pool of experts and the second approach requires extensive experimental data for calibration as well as numerical models which can represent complex failure mechanisms, the empirical approach uses actual damage data to obtain realistic fragility curves for the given study region.

Various statistical models have been used to obtain empirical fragility curves. These include parametric generalized linear models (GLMs), semiparametric generalized additive models (GAMs), and nonparametric kernel‐based methods (Lallemant, Kiremidjian, & Burton, 2015; Rossetto, Ioannou, Grant, & Maqsood, 2014). Usually, in a GLM, the log‐transformed IM is used as a covariate or independent variable and the binomial likelihood is used to describe the probabilities of damage grade exceedance given the IM value. Together with the estimated regression parameters, the choice of the link function (e.g., logit, probit, or complementary log–log), which relates the linear predictor to the probability of exceedance, determines the shape of the fragility curve.

The GAM extends the features of the GLM by allowing for nonlinear relations with the covariates through cubic smoothing splines. Due to the added flexibility, a smoothing parameter is used to avoid overfitting the data. Although less common than GLMs and GAMs, kernel‐based methods such as two‐dimensional Gaussian kernel smoothing and kernel‐weighted linear regression have also been used to derive empirical fragility curves (Noh, Lallemant, & Kiremidjian, 2015). These do not require an assumption of the shape of the fragility curve but face the issues of boundary effects (nonzero probability at zero IM) as well as overfitting which can be mitigated by zero padding and adjusting the kernel bandwidth, respectively.

In this article, we focus on empirical fragility curves based on the lognormal cumulative distribution function for multiple damage grades. While our demonstrations are in the context of earthquake hazard, the methods and conclusions can be extended to other hazards (e.g., volcanic, hurricane), to other functional forms (e.g., logistic curves) and to other data sources (e.g., analytical or expert‐based data as long as they relate IM to separate damage grades). We focus on lognormal fragility curves because they have been shown to fit well to structural and nonstructural component failure data, as well as building collapse by the incremental dynamic analysis (IDA) (Bird, Bommer, Bray, Sancio, & Spence, 2004; Bradley & Dhakal, 2008; Rossetto & Elnashai, 2005; Sarabandi, Pachakis, & King, 2004; Singhal & Kiremidjian, 1996). Their use is not only well‐established in disaster risk analysis (Kennedy, Cornell, Campbell, Kaplan, & Perla, 1980; Kennedy & Ravindra, 1984; Kircher, Nassar, Kustu, & Holmes, 1997; Lamb, Garside, Pant & Hall, 2019) but also has strong theoretical backing: it gives zero probability weight to zero and negative IM values; it is fully characterized by its first and second moments; and it imposes the minimum information, from an information theory perspective, based on these restrictions (Porter, Kennedy, & Bachman, 2007). With its mathematical property of closure under division and multiplication, the lognormal distribution family has also been successfully used to develop code‐oriented reliability metrics (Nuclear Regulatory Commission, 1983; Shinozuka, Feng, Lee, & Naganuma, 2000).

Lognormal fragility curves can be fit for each damage grade separately, for example, via a GLM with a probit link function (probit regression), or for all damage grades simultaneously via a cumulative link model (ordinal regression). In the former approach, damage grades are treated as nominal. That is, they are seen as categories which are distinct and not associated with a quantitative value or ordering. This leads to a loss of information when we construct nominal models for collapse fragility curves because they do not distinguish between data of “near‐collapse” and “no damage” but view both just as damage grades which are below collapse. In contrast, in the latter approach, the ordering of the damage grades is accounted for in the ordinal model through the numerical scale of a latent variable.

While it is common practice to take the nominal approach, we argue that damage data should be modeled as ordinal because it is treated as such during the collection of empirical data. In addition, as we will show though our analyses, it is far more beneficial to treat the damage grades as ordered categories. This leads to higher sensitivity to the overall structure of the damage grades, as well as parsimony, i.e., being able to explain the data with fewer model parameters. Associated with the latter is a lower risk of overfitting. By using an ordinal regression, we avoid the commonly faced problem associated with crossing fragility curves (Applied Technology Council, 2012). By using data across damage grades, we also obtain fragility curves with lower associated uncertainty than those obtained by fitting curves for each damage grade separately.

In Section 2, we introduce the theory behind an ordinal regression and explain its advantages over separate probit regressions. Note that the ordinal regression framework includes probit regression as a special case where we only have one damage grade. To illustrate the insensitivity of the nominal approach to distinct damage data sets, its higher risk of overfitting, and the issue of crossing fragility curves, we generate and fit fragility curves via ordinal and separate probit regressions to synthetic data sets in Section 3. Using the case study of the Nepal 2015 earthquake in Section 4, we show that the ordinal regression provide not only noncrossing fragility curves but also curves with lower associated uncertainty for real‐life damage data. Finally, in Section 5, we discuss our findings before concluding with remarks on further work in Section 6.

## 2 ORDINAL REGRESSION

### 2.1 Theory

Ordinal regressions, also known as cumulative link models, treat damage grades as ordered categories which arise due to the binning of a real‐valued latent variable (Agresti, 2002; Greenwell, McCarthy, Boehmke, & Liu, 2018; Lallemant et al., 2015). Since damage scales, like the EMS‐98 damage scale, are themselves discretized by thresholds into grades, the use of a latent variable and binning into intervals in an ordinal regression seems natural. The latent variable scale can also be seen as a transformation of the 0–100 damage scale to an unbounded, real line to enable modeling with a normal distribution.

**Fig 1:**
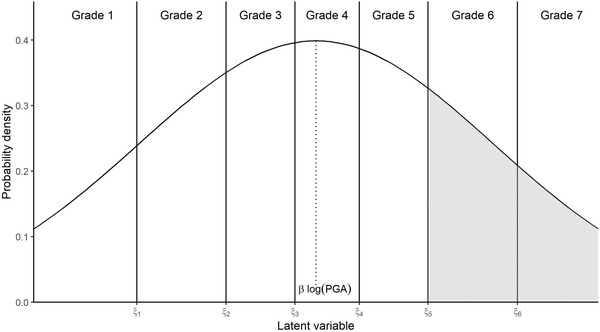
Example of the probability distribution of the latent variable $Z_{j}$ in an ordinal model. The bold vertical lines denote the cutoff points $\left\{\xi_{k}:k=1,\text{…},6\right\}$ and the dotted vertical line denotes the mean which depends on the peak ground acceleration (PGA) value. The shaded area corresponds to the probability that the building exceeds Damage Grade 6 (i.e., obtains Grade 6 or above); the unshaded area corresponds to the probability that the building is in Grade 5 or less.

Fig. 1 shows the normal distribution for the latent variable associated with a particular building j∈{1,…,J} where J is the number of buildings in our data set. Here, the x‐axis represents the latent variable scale and the bold vertical lines denote the cutoff points, $\left\{\xi_{k}:k=1,\text{…},K-1\right\}$ where K is the number of damage grades in the damage grading system. Note that $\xi_{K}=\infty$. The cutoff points demarcate the bins for the latent variable $Z_{j}$ so that when $Z_{j}$ is less than a particular cutoff point ($\xi_{k}$), the damage grade $D_{j}$ is less than or equal to the corresponding damage grade, k.

By assuming that $Z_{j}$ has normal distribution with a mean which is directly proportional to the logarithm of a hazard IM, such as the peak ground acceleration (PGA) for the seismic setting, we can calculate the probability of the building's damage grade being less than or equal to a certain grade as follows:

(1) $$\begin{array}{cc} P(D_{j}\leq k) & =P(Z_{j}\leq \xi_{k})\\ & =P(\varepsilon_{j}+\beta \log\left(PGA\right)\leq \xi_{k})\\ & =\Phi (\xi_{k}-\beta \log\left(PGA\right)), \end{array}$$

where $\varepsilon_{j}∼N\left(0,1\right)$, β∈R, and Φ is the cumulative distribution function of the standard normal distribution or the probit link. Just as how this quantity corresponds to the area under the probability density curve in Fig. 1 for $Z_{j}$ values less than $\xi_{k}$, we can calculate the probability of the building being in a certain damage grade as the area under the curve between the two adjacent cutoff points and calculate the exceedance probability of a damage grade as the area under the curve to the right of the previous cutoff. This is illustrated by the shaded area for Grade 6 in Fig. 1. Note that since higher PGA is expected to lead to greater damage, β is expected to be positive.

The likelihood of observing the entire damage data set can be defined as the joint probability of the J buildings being in their observed damage grades. This is the product of the probabilities of each individual building being in its damage grade, as follows:

(2) $$\begin{array}{ccc} L(\beta ,\left\{\xi_{k}\right\}) & = & P(D_{1}=d_{1},\text{…},D_{J}=d_{J})\\ & = & \prod_{j=1}^{J}\left(P\left(Z_{j}\leq \xi_{d_{j}}\right)-P\left(Z_{j}\leq \xi_{d_{j}-1}\right)\right)\\ & = & \prod_{j=1}^{J}(\Phi \left(\xi_{d_{j}}-\beta \log\left(PGA_{j}\right)\right)\\ & & -\Phi (\xi_{d_{j}-1}-\beta \log\left(PGA_{j}\right))) \end{array}$$

Based on the model, this is a function of the parameters β and $\left\{\xi_{k}:k=1,\text{…},K\right\}$. To fit the ordinal regression and obtain estimates of these parameters, we maximize the likelihood of the data. This is implemented in the R software using the “polr” function of the MASS package.

From Equation (1), we see that for each damage grade k, we obtain a lognormal fragility curve, which is popular among the disaster risk assessment community because it has been shown to fit failure data as well as building collapse data well, and has desirable theoretical properties (Lamb et al., 2019; Porter et al., 2007). An alternative parameterization of the model with $\tilde{\beta }=1/\beta$ as the logarithmic standard deviation of the normal distribution and $m_{k}=\exp\left(\xi_{k}/\beta \right)$ as its median value leads to the characterization of the fragility curve for the exceedance of Grade k in Porter et al. (2007) and Lallemant et al. (2015):

(3) $$F_{k}\left(PGA\right)=\Phi \left(\frac{\log(PGA/m_{k})}{\tilde{\beta }}\right).$$

For those who prefer this alternative parameterization, rewriting the likelihood in terms of $\tilde{\beta }$ and $\left\{m_{k}:k=1,\text{…},K\right\}$, we have:

(4) $$\begin{array}{ccc} L(\tilde{\beta },\left\{m_{k}\right\}) & = & \prod_{j=1}^{J}\left(\Phi \left(\frac{\log\left(m_{d_{j}}\right)-\log\left(PGA_{j}\right)}{\tilde{\beta }}\right)\right.\\ & & -\left.\Phi \left(\frac{\log\left(m_{d_{j}-1}\right)-\log\left(PGA_{j}\right)}{\tilde{\beta }}\right)\right). \end{array}$$

Rather than parameterizing cutoffs, we now parameterize via the medians of the normal distributions associated with each fragility curve.

The difference between the fragility curves obtained from an ordinal regression and separate probit regressions is the assumption that the different fragility curves share the same β value as opposed to having different β values for each fragility curve fit. This is commonly referred to as the proportional odds or parallel slopes assumption.

In the ideal scenario where we have equal number of buildings in each damage grade which are spread across the range of PGA values, the separate probit regressions and ordinal regressions should give exactly the same fragility curves and estimates if the proportional odds assumption is exactly satisfied. In practice, however, we often have unequal amounts of data across damage grades and PGA values. Through the shared β and latent variable scale, the ordinal model allows us to borrow information across damage grades as well as the PGA range because certain damage grades tend to occur within certain PGA intervals.

### 2.2 Properties

There are multiple advantages of using an ordinal regression instead of separate probit regressions to derive empirical fragility curves:

1. Higher sensitivity to the damage data across the damage grades;
2. Parsimony leading to lower risk of overfitting;
3. No crossing fragility curves for the different damage grades; and
4. Lower uncertainty associated with the derived curves.

We will illustrate the first three advantages using synthetic data in Section 3 and demonstrate the last two advantages using a real‐life damage data from the Nepal 2015 earthquake in Section 4.

Ordinal regression allows us to make use of all the data at the same time by simultaneously fitting fragility curves for all damage grades. This leads to reduced uncertainty associated with the derived fragility curves, characterized by tighter confidence bounds. In addition, by viewing the damage grades as ordered and related to each other, rather than nominal, an ordinal regression is more sensitive to the overall structure of the damage data. This results in distinct fragility curves for distinctly different data sets even in cases when a probit regression results in the same fragility curve for the two data sets because it only considers the empirical proportions above or below that damage grade and does not differentiate between the other damage grades. As a more intuitive explanation of this, nominal models for a collapse fragility curve will treat data of “near‐collapse” and “no damage” the exact same way, as both damage grades are below collapse. This is a significant loss of information.

Since it estimates one shared slope parameter, β, rather than multiple $\beta_{i}$ values for each damage grade, the ordinal regression is a more parsimonious model (having the quality of simplicity but great explanatory predictive power) for the purposes of estimating exceedance probabilities for multiple damage grades. This is confirmed in Section 3.3 where we see better test performance and greater robustness against noise in the data. With additional parameters, the separate probit regressions may appear to fit the data better. However, this nominal approach is more likely to suffer from overfitting and may lead to poorer test performance because of the higher risk of fitting to the noise.

The shared slope parameter in an ordinal regression allows it to avoid crossing fragility curves for the different damage grades. Crossing curves is widely recognized problem because this implies that there are negative probabilities for the affected damage grades. As mentioned by Porter et al. (2007), two lognormal fragility functions will necessarily cross if they have unequal β's and slope to cutoff ratios. That is, $\beta_{1}\neq \beta_{2}$ and $\xi_{1}/\beta_{1}\neq \xi_{2}/\beta_{2}$. Porter et al. (2007) suggest two methods to correct for crossing fragility curves. The first, which we will refer to as Method 1, sets the portion of the curve corresponding to the exceedance of the lower damage grade equal to that of the higher damage grade after crossing. This means that we impose zero probability for observing buildings in the lower damage grade. In Method 2, the β and cutoff values are adjusted so that all damage grades share the same β value and the adjusted curve meets the original curves at a 10% failure probability. While these post hoc strategies fix the crossing curves and have been used in practice (see, e.g., Giordano et al., 2021; Thapa, Shrestha, Lamichhane, Adhikari, & Gautam, 2020), the ordinal regression provides a solution to this issue with a stronger theoretical grounding.

## 3 SYNTHETIC DATA ILLUSTRATION

### 3.1 Motivation

To aid our illustration of the benefits of an ordinal regression over separate probit regressions, we create four synthetic data sets containing buildings with PGA values {0.1,0.4,0.7} and damage grades ranging from 0 to 5. Each data set has 100 buildings per PGA value.

**Fig 2:**
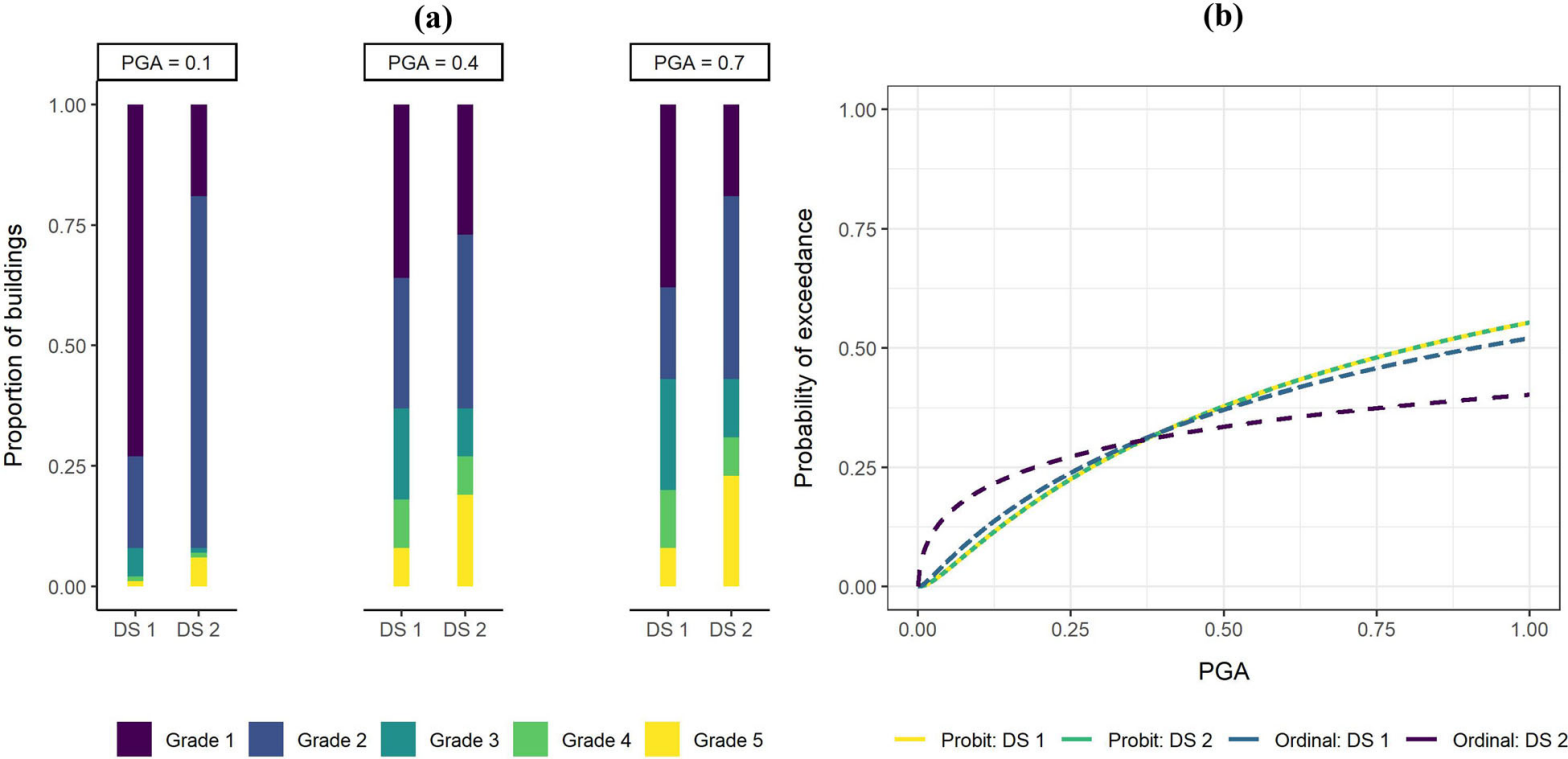
(A) Distribution of buildings according to peak ground acceleration (PGA) and damage grades in Data set (DS) 1 and 2; (B) Fragility curves derived via ordinal and probit regressions for the exceedance of Grade 3 using DS 1 and 2.

For Data set 1, the damage distribution within PGA bins varies according to an ordinal model. This is treated as a base data set. For comparison and to investigate the sensitivity of the models to the overall structure of the damage data, Data set 2 is created by switching the number of buildings per damage grade within the groups that have grades below Grade 2 and above Grade 2. The stacked barcharts in Fig. 2(A) show the damage grade proportions in Data set 1 and 2 at the three PGA values. Notice that the total proportion of buildings in Grade 1 and 2 are the same in Data set 1 and 2 but their individual proportions have been switched around. A similar operation was used to change the number of buildings for Grades 3–5.

To investigate the potential of overfitting, we add noise to Data set 1 by randomly choosing x% of the buildings and randomly changing their damage grades with a draw from the discrete uniform distribution over the damage grades (we use x=25%). This gives us Data set 3 which acts as training data. To evaluate the ordinal and probit model fits to the training data, we generate 100 test sets in the same way as Data set 1 but with different random seeds and evaluate test performance as an average over the test sets.

Data set 4 is used to investigate crossing fragility curves. The data are generated by first varying the slope parameter per fragility curve using $\beta ∼N(\hat{\beta },c\hat{\beta })$ where $\hat{\beta }$ is the slope estimate in the ordinal model used to generate Data set 1 and $c\in R^{+}$ (we use c=1.2). With the now crossing curves, we apply Method 1 from Porter et al. (2007) to obtain nonnegative damage grade probabilities which we then use to generate the damage data.

A summary of the different synthetic data sets is given in Table I.

**Table I:** Summary of the Generation Mechanisms Behind and the Uses of the Four Synthetic Data Sets

Data set	Generation	Purpose
1	Ordinal model	Base data
2	Buildings switched about Grade 2	Investigate sensitivity to data
3	Added noise to x% of damage grades	Investigate overfitting
4	Added variation to cross curves	Investigate crossing fragility curves

### 3.2 Advantage 1: Higher Sensitivity to Data

We use the ordinal and probit regressions to fit fragility curves for the exceedance of Grade 3 for Data set 1 and 2. Fig. 2(B) shows that exactly the same curve has been derived using the probit regressions for the two different data sets. This is because when we treat damage grades as nominal and only consider them one grade at a time, we are essentially converting the damage grade data into binomial data of exceedance and nonexceedance of damage. Thus, we do not make use of the information on the distribution of damage in other damage grades. In comparison, by treating damage grades as ordinal, we make full use of the data across all damage grades. Hence, the fragility curves obtained from ordinal regressions in Fig. 2(B) are different for Data set 1 and 2, as we would expect.

### 3.3 Advantage 2: Lower Risk of Overfitting

Next, we investigate the potential for overfitting using the data with added random noise (Data set 3). We use the Kullback–Leibler (KL) divergence to evaluate how well the damage grade probabilities obtained from the fitted ordinal and probit models describe the test data. This quantifies the difference in the conditional distributions of the damage grades given the PGA value, and not the difference in the fragility curves themselves. By working with the estimated distributions directly instead of damage grade classifications, we do not need to decide on a classification criteria. This also bypasses the problem faced by standard classification error of not being able to quantify the difference between for example, estimating Grade 3 for a Grade 4 building and estimating Grade 1 for the same building. For test set i∈{1,…,100}, denoted as $T_{i}$, the KL divergence at PGA = $PGA_{j}$ is defined as:

(5) $$\begin{array}{cc} & KL(T_{i},PGA_{j})\\ = & \sum_{k=1}^{K}q_{k}\left(T_{i},PGA_{j}\right)\log_{2}\left(\frac{q_{k}\left(T_{i},PGA_{j}\right)}{p_{k}\left(PGA_{i}\right)}\right), \end{array}$$

where $\left\{q_{k}\left(T_{i},PGA_{j}\right):k=1,\text{…},K\right\}$ denotes the damage grade proportions for the K damage grades from test set i at PGA$=PGA_{j}$ and $\left\{p_{k}\left(PGA_{j}\right):k=1,\text{…},K\right\}$ denotes the damage grade probabilities estimated from the probit or ordinal model fitted to Data set 3. Recall that Data set 3, our training data, is adapted from the original Data set 1 by replacing the damage grades of x=25% of its buildings by that from a uniform distribution. It is therefore a noisy version of the base data. By averaging the values of $KL(T_{i},PGA_{j})$ over 100 test sets, we obtain a measure of the goodness of fit of the model at PGA$=PGA_{j}$.

Table II shows the mean KL divergence values at the three PGA values from 100 test sets (generated from the original ordinal model with a different random seed from Data set 1) as well as their averages for the probit and ordinal models. Since its KL divergence value is lower at all the PGA values, this means that the ordinal model provides a better fit to the test data since the estimated damage grade proportions from the model is more similar to the empirical proportions.

**Table II:** Kullback–Leibler (KL) Divergence Values Comparing the Estimated Damage Grade Proportions from the Ordinal and Probit Regressions to the Empirical Proportions from Test Data at the Three Peak Ground Acceleration (PGA) Values

Model	PGA = 0.1	PGA = 0.4	PGA = 0.7	Average
Probit	0.162	0.040	0.062	0.088
Ordinal	0.132	0.039	0.036	0.069

*Note*: The mean results from 100 randomly generated test sets are shown.

**Fig 3:**
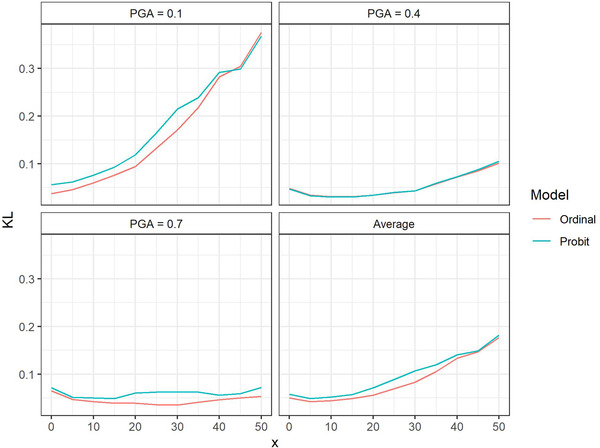
Kullback–Leibler (KL) divergence values corresponding to the probit and ordinal model predictions for different percentages of the data replaced by uniformly distributed damage grades (x). The results are shown for the three peak ground acceleration (PGA) values and averaged over the PGA values. Each KL value is computed using 100 independent test sets.

Fig. 3 shows the behavior of the KL values as a function of the amount of noise added to the data. In general, we see that the ordinal model gives lower KL values than the separate probit models. However, the extent of this difference as well as the increase in KL as noise increases differs according to the PGA value. There also seems to be a limit of the advantage of the ordinal over the probit model which is exemplified by the switch in order of KL around x=44% for PGA=0.1. This makes sense since when a large proportion of the training data is noise, neither model can reflect the original model and hence test data as well.

### 3.4 Advantage 3: Noncrossing Fragility Curves

**Fig 4:**
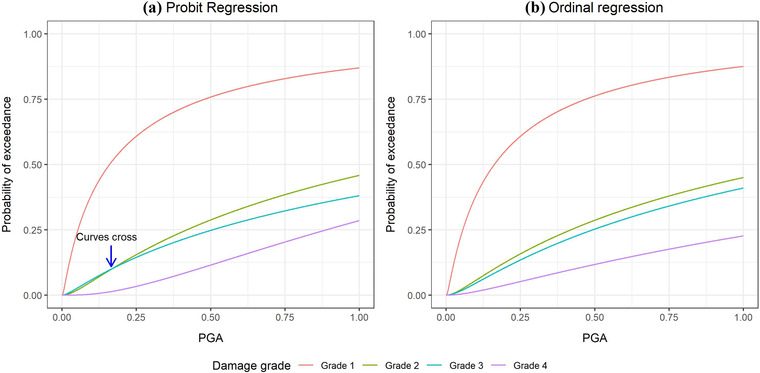
Fragility curves fitted to Data set 4 via (A) probit and (B) ordinal regressions. The blue arrow in plot (A) shows where the curves derived from the probit model cross.

In this subsection, we illustrate the issue of crossing fragility curves seen in the nominal approach using Data set 4. We fit probit regressions for each damage grade and compare the results to that from the ordinal regressions. Fig. 4 shows that the curves from the separate probit regressions cross while those from the ordinal regressions do not. This will be illustrated again using the case study in the next section.

## 4 CASE STUDY: NEPAL 2015 EARTHQUAKE

To further illustrate the benefits of ordinal regression over separate probit regressions, we use building damage data collected in the aftermath of the magnitude 7.8 Gorkha earthquake in Nepal on April 25, 2015. The Government of Nepal commissioned the Earthquake Housing Damage and Characteristics Survey to conduct field surveys to classify buildings according to the EMS‐98 damage grading system (Kathmandu Living Labs, 2019; Lallemant et al., 2017; Tyagunov, Grünthal, Wahlström, Stempniewski, & Zschau, 2006). Data were gathered from the 11 districts most affected by the earthquake.

For our demonstration, we focus on buildings with timber superstructure on flat ground. Of the 20,043 buildings surveyed, 8,891 were in Damage Grade 1 (negligible to slight damage) with central damage factor (CDF) 0.5, 4,325 were in Grade 2 (CDF =10), 3,437 were in Grade 3 (CDF =40), 1,719 were in Grade 4 (CDF =80), and 1,671 were in Grade 5 (CDF =100; collapse). There were no buildings in Damage Grade 0 (CDF =0; no damage). This reflects both reality and the sample design since the damage survey only covered the 11 districts most affected by the earthquake and the threshold between Grade 0 and Grade 1 was very small (a hairline crack would be classified under Grade 1). Since the locations of the buildings are known up to the ward‐level, the centroids of the wards were used to extract the corresponding PGA values obtained from the USGS ShakeMap (United States Geological Survey, 2020; Worden & Wald, 2016).

**Fig 5:**
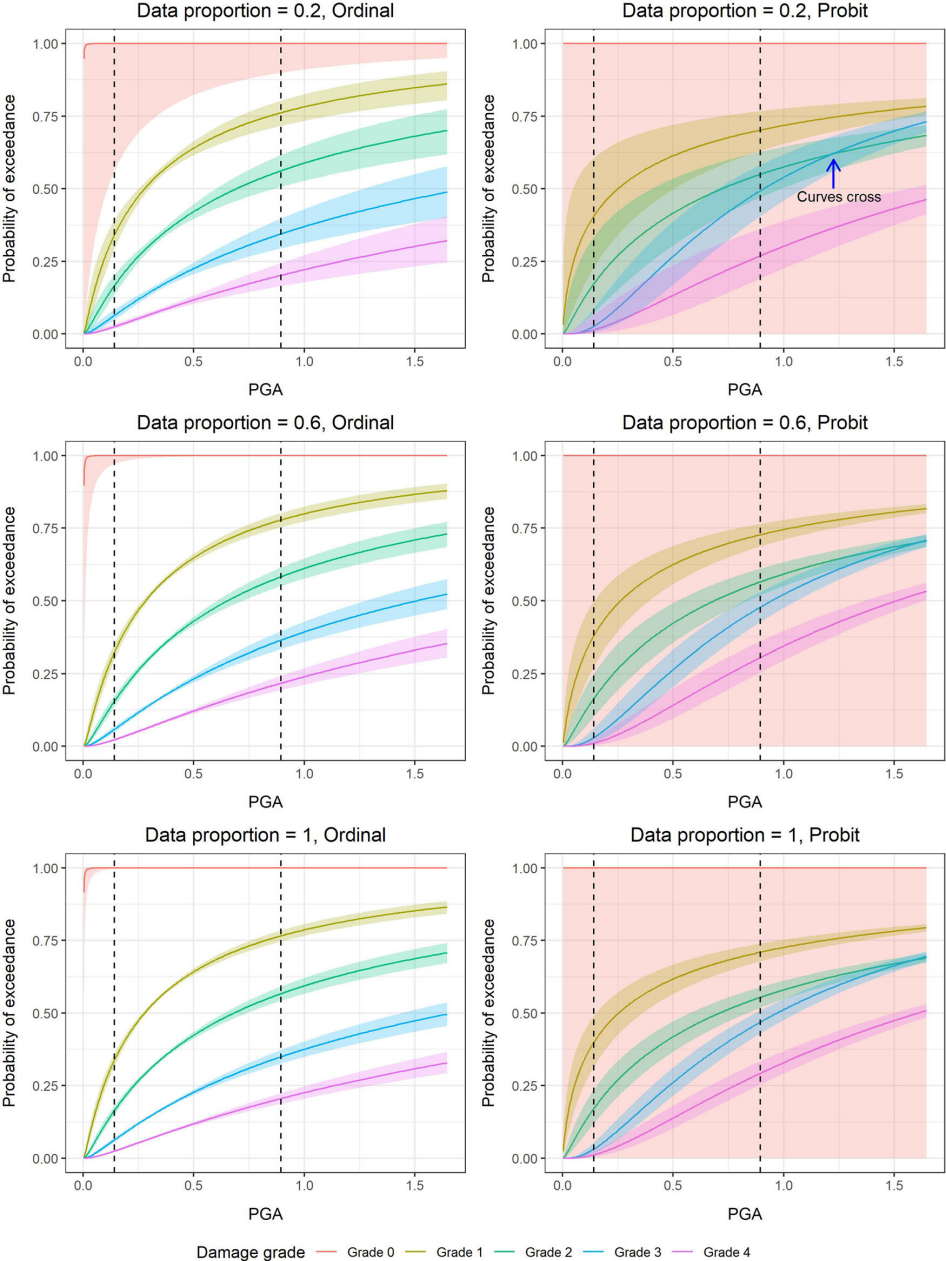
Fragility curves obtained from fitting ordinal and separate probit regressions to 20%, 60%, and 100% of the Nepal damage data. The colored bands correspond to the 95% confidence intervals. The black vertical dotted lines denote the PGA range covered in the data. The blue arrow in the top right plot shows where the curves derived from the probit regressions cross when the data proportion is set to 0.2.

Fig. 5 shows the fragility curves derived from ordinal regressions (first column) and separate probit regressions (second column) together with their 95% confidence intervals (CIs). An increasing proportion of the data (20%, 60%, 100%; taken proportionally across damage grades) is used to derive the curves on the first to the third rows and the 95% CI bounds are calculated using the analytical formulas derived in Lallemant and Burton (2020). Different proportions of the data were considered to show the effect of sample sizes on the fragility curves and their uncertainties.

Focusing on the red curves and uncertainty envelopes, we note that in comparison to the ordinal regression, the probit regression gives extremely wide CIs for the fragility curve describing the exceedance of Damage Grade 0. This is because there are no Grade 0 buildings and the corresponding probit regression acts as a dummy model. Despite the lack of data for this damage grade, the ordinal regression is able to give sensible estimates by borrowing information from data on other damage grades and the CIs of its fragility curves narrow when more and more data are used. The ordinal model consistently gives estimates with lower uncertainties than the probit regressions since it makes better use of all damage grade data.

**Fig 6:**
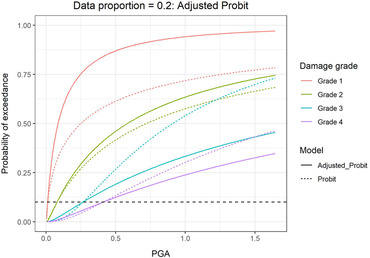
Fragility curves for Grades 1–4 obtained from the adjusted and original probit regressions. The horizontal black dotted line denotes 10% failure probability.

In addition to lower uncertainties, the fragility curves derived from the ordinal regression are preferable to those from separate probit regressions because they do not cross. The crossing of curves from the probit regressions is seen most clearly when 20% of the data is used for fitting (top right plot of Fig. 5). As mentioned before, Porter et al. (2007) suggest two methods to correct for crossing fragility curves. We illustrate Method 2 where the β and cutoff values are adjusted so that all damage grades share the same β value and the adjusted curve meets the original curves at a 10% failure probability. This is shown in Fig. 6 for case where 20% of the damage data is used. Although it has been observed that building component reliability is insensitive to β when the engineering demand parameter (EDP) at 10% failure probability is established (Kennedy & Short, 1994), the choice of fixing the curves at 10% failure probabilities is still somewhat subjective. Furthermore, by construction, the ordinal model will give fragility curves which fit the data better than the nominal approach with Method 2 adjustment. In this case, the ordinal model gives an Akaike information criterion (AIC) value of 11,246 which is lower than 13,000, obtained for the adjusted probit models. This indicates that the ordinal model provides better fit.

## 5 DISCUSSION

Empirical fragility curves based on the lognormal cumulative distribution function are popular because they have desirable theoretical properties and have been shown to fit various types of damage data well. In Section 2, we introduced the theory of ordinal regression, a model which provides lognormal fragility curves for all the damage grades in the data simultaneously. This relies on a continuous latent variable which is discretized into discrete bins in similar fashion to how damage scale is discretized into damage grades in practice. Through this latent variable, the natural ordering of the damage grades, which damage surveyors consider in their evaluation of buildings, is preserved.

As we have illustrated in Sections 3 and 4 through synthetic data and real‐life damage grade from the Nepal 2015 earthquake, there are multiple advantages of incorporating this ordering of damage grades in the damage model and interpreting the grades as ordinal rather than nominal. These are summarized in Table III.

**Table III:** Advantages and Disadvantages of Using Ordinal Regressions and Separate Probit Regressions for Fitting Empirical Fragility Curves

Model	Advantages	Disadvantages
Ordinal	Higher sensitivity to damage data across grades. Parsimony and lower risk of overfitting. Noncrossing fragility curves. Curves with lower uncertainty. Can give sensible results in the absence of data for certain damage grades. Fits curves for all damage grades simultaneously.	Proportional odds assumption. Can seem like poorer fit to training data.
Probit	Can seem like better fit to training data.	Lower sensitivity to damage data across grades. More parameters and higher risk of overfitting. Crossing fragility curves. Curves with higher uncertainty. Results not sensible in the absence of data for damage grade. Fits curves for each damage grade separately.

A common reason given for favoring the nominal approach of fitting separate probit regressions for each damage grade is that the derived fragility curves seem to fit the data better with visual inspection. This is due to the greater number of parameters involved in the combined models: while the ordinal model uses one slope parameter (β) across damage grades, the nominal approach estimates one slope parameter per damage grade. This seemingly better fit, however, comes at the expense of overfitting. In the case of noisy data with measurement error, the models can fit to the noise instead of the signal, i.e., the relation between the ground motion IM and building damage. This results in poorer out‐of‐sample prediction of the damage grade distributions despite better training performance as illustrated in Section 3.3.

Another problem that arises in the nominal approach is crossing fragility curves which imply the existence of zero and negative damage grade probabilities as seen in both Sections 3.4 and 4. As seen in the latter section, fixing this issue through *post hoc* strategies such that those suggested in Porter et al. (2007) leads to poorer fit to the data than that achieved by the ordinal regression.

From the analyses, it seems that the disadvantages of the nominal approach outweigh its advantages. There are also clear benefits of using the data across damage grades together through an ordinal regression. In Section 3.2, we saw that the ordinal approach is able to differentiate between Data sets 1 and 2 via the fragility curves for the exceedance of Grade 3. In comparison, the nominal approach was insensitive to these different damage data sets. Second, by making use of all the damage data, the fragility curves derived through ordinal regression have lower associated uncertainty than those obtained from separate probit regressions. In addition, by borrowing information across the damage grades, the ordinal model can provide sensible estimates in the absence of data for a certain damage grade while the nominal approach cannot. Another advantage of the ordinal approach is the convenience of one model optimization for all the fragility curves. This stands in contrast to the need to perform multiple optimizations in the nominal approach and use *post hoc* adjustments to ensure that the results are consistent with each other.

## 6 CONCLUSION AND FURTHER WORK

Following a disaster, empirical damage grade data are gathered by evaluators who assign ordinal measurements to damage characteristics. Although we presented our analyses in the context of earthquake hazard, the same issues and proposed ordinal model solution applies to other hazards. For example, for typhoons, one might use wind speed instead of PGA as a predictor variable. While we have focused on empirical fragility curves, the ordinal and probit models can also be used to fit expert‐based and analytical fragility curves that use limit states to define damage.

Through our comparison of the two models, we demonstrate that treating damage grades as ordinal in the development of fragility curves provides numerous advantages. Compared to the nominal approach of fitting separate lognormal fragility curves for each damage grade, the ordinal model provides a more comprehensive and realistic representation of building damage and the natural order of the discrete damage grades. As illustrated through our analyses, there are multiple advantages of making this ordering explicit in the damage model including noncrossing fragility curves, lower curve uncertainty, less overfitting, better out‐of‐sample predictive power, and more.

The issue of crossing fragility curves obtained from multiple probit regressions for different damage grades is widely known yet a vast majority of studies do not address this at the modeling stage and either ignore it completely or demonstrate that it does not occur in the hazard intensity ranges that they are interested in. Recently, however, there are studies using ordinal regressions (see, for example, Charvet, Suppasri, and Imamura (2014) and Charvet, Macabuag, and Rossetto (2017)) and new models which do account for damage grade ordering. For example, Andriotis and Papakonstantinou (2018) propose the use of softmax regressions to allow some parametric flexibility for fragility curves beyond the classical lognormal assumption. Damage grade ordering can be enforced by constricting the separating hyperplanes to be parallel (the ordinal approach) or by modeling exceedance probabilities for one damage grade conditional on lower damage grades (the hierarchical approach). The ordinal softmax regression can be seen as an extension of the ordinal regression presented in this article.

Another extension of the ordinal regression which has been used recently is the multinomial logistic regression with the constraint of ordered damage grade specific intercepts (Yazdi, Haukaas, Yang, & Gardoni, 2016). In addition to avoiding crossing fragility curves, this allows for the identification of additional variables that affect damage (on top of the single hazard IM).

As per convention, the fragility curves we examined are univariate in that they only account for the relationship between building damage and one hazard IM. In this article, we used the PGA as a ground motion IM. This describes the high‐frequency motion that the short and stiff buildings are sensitive to. While such buildings form the majority in Nepal, our case study, there are other important aspects of ground motion such as duration and frequency content which affect building damage. Incorporating additional factors in the damage model is an active area of research in disaster risk analysis. For coastal floods, a three‐dimensional loading parameter space has been proposed to consider the effects of water level, wave height, and period on failure probability (Jane et al., 2018). In the seismic setting, a spatial ordinal model is currently in development to address the additional IMs as well as other factors which influence the spatial correlation in building damage such as local soil conditions and similarities in construction age and material.

This article provides one of the first formal demonstrations of multiple advantages of the ordinal model over the nominal approach. With this, we hope to encourage the further use and development of these ordinal and hierarchical ideas to model damage grade distributions.

## DATA AND CODE AVAILABILITY

The R code for the generation and analysis of the synthetic data as well as Nepal damage data can be found at https://github.com/ntu‐dasl‐sg/ordinal_fragility_public. The Nepal damage data and Shakemap are publicly available from Kathmandu Living Labs (2019) and United States Geological Survey (2020).
